# Brain and Neuroscience Advances 2023

**DOI:** 10.1177/23982128231167484

**Published:** 2023-04-02

**Authors:** Kate Baker

**Affiliations:** 1 MRC Cognition and Brain Sciences Unit, University of Cambridge, Cambridge, UK; 2 Department of Medical Genetics, University of Cambridge, Cambridge, UK

*Brain and Neuroscience Advances* was launched in 2017, and pledged to be the journal which ‘puts the goals of researchers first, welcomes submissions from all areas of basic, clinical, and translational neuroscience’ (Aggleton and Dalley, 2017). As the society journal of the British Neuroscience Association, this is a publication which celebrates diverse neuroscience and neuroscientists, providing a ‘forum for debate and progress’ as well as a solid platform for disseminating high-quality empirical studies.

So what’s happened since 2017? *Brain and Neuroscience Advances* has achieved its aims by publishing highly cited papers across clinical, cognitive, systems and molecular neurosciences. The journal has championed best practice in publication ethics, and is a beacon for open science innovations including registered reports. The journal has remained true to its ethos of being responsive to its authors and audience, introducing new article formats such as a Journal Club, and commissioning Special Collections in partnership with field-leading and early-career researchers.

Between 2017 and 2022, there has been no shortage of opportunities and challenges for the journal. Neuroscience continues to draw-in ever more students and scientists from psychology and biological sciences, with increasing representation from engineering, physics and computer sciences. A challenge is communicating across scientific languages to develop integrated, useful models of brain function in health and disease – a challenge which this journal can rise to by publishing work across methodologies and topics. The COVID pandemic hit neuroscience hard – it was a wake-up call for brain health (acutely in the context of severe viral infection, chronically in the context of rehabilitation and recovery), and alerted us to the vulnerability of research and researchers during unexpected crises. Meanwhile science funding and career structures have become more uncertain and unstable, and the publication landscape has become chaotic and complex to navigate. *Brain and Neuroscience Advances* has held steady during these storms, recognising that affordable peer-reviewed open access publication remains the bedrock on which neuroscientific knowledge and careers can flourish.

And what’s new in 2023? I have joined Jeff Dalley as co-editor-in-chief, bringing some new perspectives to the journal’s strong and creative leadership. I am a clinician by training and practice, and a developmental cognitive neuroscientist. My own research spans genomics, neurodevelopmental disorders and mental health, and I like reading and writing across neuroscience topics and methods. Publishing articles can be a confidence-testing process – I will be a friendly, approachable and supportive editor, and I look forward to publishing your work in *Brain and Neuroscience Advances* so that the journal and field continue to thrive.
